# A Retrospective 2D Morphometric Analysis of Adult Female Chiari Type I Patients with Commonly Reported and Related Conditions

**DOI:** 10.3389/fnana.2018.00002

**Published:** 2018-01-19

**Authors:** Maggie S. Eppelheimer, James R. Houston, Jayapalli R. Bapuraj, Richard Labuda, Dorothy M. Loth, Audrey M. Braun, Natalie J. Allen, Soroush Heidari Pahlavian, Dipankar Biswas, Aintzane Urbizu, Bryn A. Martin, Cormac O. Maher, Philip A. Allen, Francis Loth

**Affiliations:** ^1^Department of Biomedical Engineering, Conquer Chiari Research Center, University of Akron, Akron, OH, United States; ^2^Department of Psychology, Conquer Chiari Research Center, University of Akron, Akron, OH, United States; ^3^Department of Radiology, University of Michigan Health System, Ann Arbor, MI, United States; ^4^Conquer Chiari, Wexford, PA, United States; ^5^Department of Mechanical Engineering, Conquer Chiari Research Center, University of Akron, Akron, OH, United States; ^6^Duke University Medical Center, Duke Molecular Physiology Institute, Durham, NC, United States; ^7^Department of Biological Engineering, University of Idaho, Moscow, ID, United States; ^8^Department of Neurosurgery, University of Michigan Health System, Ann Arbor, MI, United States

**Keywords:** Chiari I malformation, morphometry, MRI, posterior cranial fossa, cranio-vertebral junction, comorbidity, prevalent conditions, related conditions

## Abstract

**Purpose:** Researchers have sought to better understand Chiari type I malformation (CMI) through morphometric measurements beyond tonsillar position (TP). Soft tissue and bone structures within the brain and craniocervical junction have been shown to be different for CMI patients compared to healthy controls. Yet, several morphological characteristics have not been consistently associated with CMI. CMI is also associated with different prevalent conditions (PCs) such as syringomyelia, pseudotumor, Ehlers-Danlos syndrome (EDS), scoliosis, and craniocervical instability. The goal of this study was two-fold: (1) to identify unique morphological characteristics of PCs, and (2) to better explain inconsistent results from case-control comparisons of CMI.

**Methods:** Image, demographic, and PC information was obtained through the *Chiari1000*, a self-report web-accessed database. Twenty-eight morphometric measurements (MMs) were performed on the cranial MR images of 236 pre-surgery adult female CMI participants and 140 female healthy control participants. Custom software was used to measure 28 structures within the posterior cranial fossa (PCF) compartment, craniocervical junction, oral cavity, and intracranial area on midsagittal MR images for each participant.

**Results:** Morphometric analysis of adult females indicated a smaller McRae line length in CMI participants with syringomyelia compared to those without syringomyelia. TP was reduced in CMI participants with EDS than those without EDS. Basion to posterior axial line was significantly longer in CMI participants with scoliosis compared to those without scoliosis. No additional MMs were found to differ between CMI participants with and without a specific PC. Four morphometric differences were found to be consistently different between CMI participants and healthy controls regardless of PC: larger TP and a smaller clivus length, fastigium, and corpus callosum height in CMI participants.

**Conclusion:** Syringomyelia, EDS, and scoliosis were the only PCs that showed significant morphometric differences between CMI participants. Additionally, four midsagittal MR-based MMs were found to be significantly different between healthy controls and CMI participants regardless of the presence of one or more PCs. This study suggests that the prevalence of comorbid conditions are not strongly related to CMI morphology, and that inconsistent findings in the radiographic literature cannot be explained by varying prevalence of comorbid conditions in CMI study samples.

## Introduction

Chiari type I malformation (CMI) is a complex neurological malformation within the brain that is diagnosed by a tonsillar position (TP) of at least five millimeters below the foramen magnum (FM), also known as tonsillar ectopia (TE), in conjunction with a myriad of symptoms, including headache, paresthesia, gait disturbance, and sleep apnea amongst others. Prevalence within the United States has been estimated to range from 0.6 to 1%, where this rate varies depending on diagnostic criteria (Meadows et al., 2000; Speer et al., 2000; Strahle et al., 2011; Smith et al., 2013). In contrast, TE is found in 1–2% of individuals without a CMI diagnosis, suggesting that TP is limited as a reliable marker of CMI status (Smith et al., 2013). Therefore, it is advantageous to explore the utility of alternative morphological markers to distinguish symptomatic CMI patients from healthy individuals. Previous studies have found associations with CMI and bone structures of the posterior cranial fossa/skull base, and the craniocervical region such as basilar invagination, small posterior cranial fossa (PCF), abnormal segmentation of the odontoid process, and a short clivus bone (Sahuquillo et al., 1994; Nishikawa et al., 1997; Milhorat et al., 1999, 2010; Besachio et al., 2015; Houston et al., 2017). These differences in soft tissue and bony structures within the PCF and upper cervical spinal canal have been suggested to result in symptoms related to lower brainstem and upper cervical cord compression (Sinclair et al., 2002).

It has also been suggested that soft tissue and bone structures within the brain and craniocervical junction vary between CMI patients presenting with differing etiologies of CMI due to different TE mechanisms. Conditions related to cranial settling, intracranial hypertension, and spinal cord tethering have been previously characterized as TE mechanisms (Milhorat et al., 2010). These mechanisms can be categorized as having either a causative or consequential relationship with TE. Causative factors are those that disturb the migration of the spinal cord during development or result in cranium malformations in the bones, particularly those in the PCF. Consequences of TE are those which result in compression of the brain stem by bony elements or TE (deSouza et al., 2011). Conditions that are commonly related to CMI such as syringomyelia, pseudotumor cerebri, Ehlers-Danlos syndrome (EDS), scoliosis and craniocervical instability have been classified as a TE mechanism through either a causative or consequential relationship with TE (Milhorat et al., 2010; deSouza et al., 2011; Klekamp, 2012). Additional diseases such as cystic fibrosis and fibromyalgia have been previously reported as diagnosed alongside CMI but not considered a TE mechanism (Urbizu et al., 2017b).

While they provide a better understanding of CMI, there are a limited number of studies that have examined morphological distinctiveness of prevalent conditions comorbid to CMI (see Table 1). For example, Milhorat et al. (2007) evaluated CMI patients with hereditary disorders of connective tissue (HDCT) and found increased retro-odontoid pannus formation to be unique in CMI patients with HDCT such as EDS when compared to CMI patients without HDCT. In another example, Roller et al. (2015) found unique morphology for CMI patients with pseudotumor when compared to CMI patients without pseudotumor and healthy controls. Yet, apart from these and similar examples, the understanding of the brain morphologies of conditions related to CMI are largely limited.

**Table 1:** Descriptions of prevalent conditions.

**Condition**	**Description**	**Citations**
Syringomyelia (SM)	Development of a fluid-filled cyst in the spinal cord that has been attributed to a smaller posterior cranial fossa.	Sahuquillo et al., 1994 Depreitere et al., 2000 **Yan et al.**, **2016** **Thompson et al.**, **2016**
Pseudotumor cerebri	Syndrome associated with elevated intracranial hypertension (ICP) with no radiographic evidence of a mass or lesion in the brain. Pseudotumor cerebri has been postulated to be due to an obstruction of CSF flow at the FM and a smaller PCF volume. Pseudotumor cerebri is also known as idiopathic intracranial hypertension.	Banik et al., 2006 Galgano and Deshaies, 2013 **Roller et al.**, **2015**
Ehlers-Danlos syndrome (EDS)	A condition that encompasses a wide variety of connective tissue disorders that have an association with CMI. Such disorders include joint hypermobility, recurrent joint dislocations, poor wound healing, tendon and muscle rupture, vascular fragility, easy bruising, and arterial rupture.	**Milhorat et al.**, **2007**
Scoliosis	Characterized by an abnormal curvature or rotation of the spine. Spine curvature direction has been associated with asymmetrically displaced tonsils. A co-existing diagnosis of scoliosis with CMI has shown an atypical curve compared to idiopathic scoliosis. Surgical intervention for CMI has been associated with the stabilization of spinal curvature.	**Zhu et al.**, **2016** Brockmeyer et al., 2003
Craniocervical instability (CCI)	Classified as excessive motion between adjacent cervical vertebrae. CCI may result from abnormal development of bone or ligaments, or increasing ligamentous laxity associated with connective tissue disorders. Symptoms include neck pain or weakness in the neck.	Steilen et al., 2014 Wills and Dormans, 2006
Chronic fatigue syndrome (CFS)	A syndrome characterized by debilitating fatigue for more than 6 months. Frequent symptoms include muscle pain, multi-joint pain, and impaired memory or concentration.	Garland and Robertson, 2001
Fibromyalgia	A condition characterized as chronic body-wide pain with the absence of an inflammatory response or a degenerative musculoskeletal disorder. Patients diagnosed with fibromyalgia exhibit additional symptoms similar, or identical to, those characterized by CMI diagnosis.	Garland and Robertson, 2001 Heffez et al., 2004 **Watson et al.**, **2011**
Raynaud phenomenon	A nerve disease which occurs due to episodic vasospasms of the finger and toes when exposed to cold temperatures.	Block and Sequeira, 2001
Migraine headaches	Poorly understood phenomena consisting of recurring severe headaches and vision changes. Migraine headaches in CMI patients have been postulated to result from craniospinal pressure dissociation and dysfunction within the brainstem.	Kaplan and Oksuz, 2008
Sleep Apnea	A sleep related disorder which presents as either central or obstructive. Central sleep apnea occurs when the respiratory center drive fails to operate. Obstructive sleep apnea occurs due to closure of the pharyngeal airway caused by suction pressure that is created during inspiration and inadequate muscle tone.	Arcaya et al., 1993

*CMI morphometric studies are bolded*.

Due to a high prevalence of additional diagnoses alongside CMI in most case-control studies, it is difficult to identify morphological characteristics that are unique to CMI patients rather than morphological differences that are driven by additional co-existing conditions. For example, previous case-control studies have identified smaller PCF morphology and larger FM diameters in CMI patients compared to controls (Milhorat et al., 1999; Karagöz et al., 2002; Aydin et al., 2005; Dagtekin et al., 2011; Alperin et al., 2015). In these case-control studies, more than 50% of the CMI patients were also diagnosed with syringomyelia. Such studies make it difficult to morphologically distinguish between CMI patients with and without co-existing conditions.

In an effort to establish sub-groups of CMI patients based on etiological factors, Milhorat et al. (2010) examined the brain morphologies of CMI patients with different comorbid conditions. Their analyses resulted in the creation of three etiological sub-groups. These subgroups were: (1) classical CMI with no etiological cofactors, (2) CMI associated with HDCT, occipitoatlantoaxial joint instability and cranial settling, and (3) CMI associated with hydrocephalus and intracranial mass lesions. While multiple subgroups were evaluated against healthy controls, unique morphological characteristics were only identified for classical CMI patients. Apart from Milhorat et al. (2010), to our knowledge, no additional large-scale comparison (i.e., *n* > 300) of CMI prevalent conditions has been published in the literature.

The goal of this study was to identify morphological characteristics that are unique to five conditions associated or related to CMI. These conditions include syringomyelia, pseudotumor cerebri, EDS, scoliosis, and CCI. These five related conditions (RCs) were selected because of their association with CMI and prevalence within our dataset. Seven additional prevalent conditions (APCs) in our dataset were also examined in this study. Thus, in total we examined the morphological distinction of these 12 prevalent conditions (PCs), which consists of five RCs and seven APCs. Cranial morphological measurements were evaluated for the 12 PCs in a large sample of adult female CMI participants. The measurements for CMI participants with a specific PC were compared to CMI subjects without that specific PC. In addition, the measurements for CMI participants with a specific PC were compared to healthy controls. Furthermore, the PCs were stratified into groups similar to those defined by Milhorat et al. (2010) in order to compare morphometric measurement (MM) findings. Based on the few previous findings of morphological differences between CMI participants with and without PCs, we predict that bone morphology will differ in participants with connective tissue disorders such as EDS (see Milhorat et al., 2007). These morphologies may include a narrower odontoid angle, a wider dural angle, a larger anteroposterior dura-opisthion diameter, a larger Grabb-Oakes, or a larger basion to posterior axial line (PAL). Additionally, we believe that by comparing CMI groups with various PCs to healthy control participants, we may gain a better understanding of the inconsistent findings of case-control comparisons in CMI (Nishikawa et al., 1997; Milhorat et al., 1999, 2010; Karagöz et al., 2002; Aydin et al., 2005; Alperin et al., 2015).

## Methods

### Ethics statement

This study was approved by the local institutional review board of The University of Akron. All authors declare that they have no competing interest.

### Participants

MR images from pre-surgery CMI adult females (*n* = 236) and healthy controls (*n* = 140) were evaluated. Adult women were the primary focus of this analysis because a large majority of participants of the *Chiari1000* were adult female. RCs within our sample consisted of syringomyelia (*N* = 38), pseudotumor cerebri (*N* = 20), EDS (*N* = 21), scoliosis (*N* = 45), and CCI (*N* = 17). APCs include CFS (*N* = 31), fibromyalgia (*N* = 33), Raynaud phenomenon (*N* = 18), migraine headaches (*N* = 147), spinal dysraphism (*N* = 29), other endocrine diseases (*N* = 20), and sleep apnea (*N* = 16). Identification of the presence of PCs (RCs and APCs) was obtained through self-reported clinical diagnosis from the *Chiari1000* project.

Prevalence and demographic information for each RC and APC within our dataset are shown in Table 2. PCs with less than a 7% prevalence are not listed in Table 2. These PCs include: attention deficit, seizures, brain tumor, empty sella syndrome, stroke, basilar invagination, hydrocephalus, systemic lupus erythrematosis, tethered cord syndrome, Meniere's disease, atlantoaxial assimilation, multiple sclerosis, neurofibromatosis (excluding type I), connective tissue disorder (excluding EDS, Marfan, and MASS syndrome), growth hormone deficiency, abnormal pituitary, development delays, epilepsy, hyperactivity disorder, rickets, cystic fibrosis, achondroplasia, and concussion with internal bleeding. PCs presented in the table may have been identified at any point in the participant's life. Note, the PC termed “spinal dysraphism” exclude atlantoaxial assimilation, Klippel-Feil syndrome, and scoliosis. The PC termed “other endocrine diseases” excludes growth hormone deficiency, empty sella syndrome, and abnormal pituitary. PC denoted as seizures is a symptom where the cause of the seizures was not specified.

**Table 2:** Prevalence of conditions in CMI sample with demographics.

**Prevalent condition**	***N***	**Condition prevalence (%)**	**Mean age in years (*stdev*)**	**Percent caucasian (%)**
Migraine headaches	147	62	36 *(10)*	91.2
**Scoliosis**	45	19	35 *(10)*	95.5
**Syringomyelia**	38	16	32 *(10)*	92.1
Fibromyalgia	33	14	37 *(10)*	93.9
Chronic fatigue syndrome	31	13	38 *(11)*	87.1
Spinal dysraphism	29	12	35 *(10)*	93.1
**Ehlers Danlos syndrome**	21	9	36 *(10)*	100.0
Other Endocrine Diseases	20	8	37 *(11)*	95.0
**Pseudotumor cerebri**	20	8	36 *(12)*	85.0
Raynaud phenomenon	18	8	38 *(10)*	94.4
**Craniocervical instability**	17	7	38 *(10)*	88.2
Sleep Apnea	16	7	41 *(12)*	93.8

*Total CMI participants in study: 236. Conditions in bold are labeled as RC. Overall racial and ethnic prevalence in our sample: 2% Native American/Alaska Native, 2% Asian, and 6% Black/African American. PCs include some conditions that were diagnosed as a child*.

Consent for anonymized use of demographic data, health-related data, and MR images were provided by participants who contributed to the *Chiari1000* project. The *Chiari1000* project was created at The University of Akron in 2015 as a web-accessed database that obtains and stores participant information such as self-reported diagnosis, symptomatology, and cognitive and socio-emotional characteristics. Images were provided through online sharing (e.g., Dropbox), direct mail, or through participant release. An OsiriX DICOM PACS server (Pixmeo SARL, Geneva, Switzerland) was used to store anonymized MR images at The University of Akron. Inclusion of CMI participants was based on diagnosis of CMI by a physician and pre-surgical status of participant images. Pre-surgical status was determined using volunteered surgical and radiology records, patient disclosure, and inspection of MR images. CMI participants were excluded when surgery status could not be ascertained. Healthy female MR images and demographic information were obtained through the University of Minnesota, Washington University, Oxford University Human Connectome Project consortium (Van Essen et al., 2012), and Akron General Medical Center. No control participants presented with conditions that are known to affect brain morphology and or any other chronic illnesses.

### MRI image selection

DICOM MR images from 1.5T and 3T scanners that had been collected in the process of medical treatment were submitted by CMI patient participants to the *Chiari1000* project. MMs were taken by four operators using participants' midsagittal MR images. Operators were blinded to CMI diagnosis. Midsagittal selection by each operator was standardized across all participants and completed through a visual inspection of specific morphological structures in each image to mitigate morphological changes due to head tilt. Visual inspection criteria consisted of the visibility of three out of four structures in the midsagittal plane: (1) the genu of the corpus callosum, (2) the splenium of the corpus callosum, (3) the pituitary infundibulum, and (4) the cerebral aqueduct. Images that failed to meet these criteria were excluded from further analysis. Where available, measurements were taken on T1-weighted images. As a result, measurements from all but ten participants were taken on a T1-weighted image. The measurements from the remaining ten participants were taken from a T2-weighted image.

### Measurement software

**Figure 1:**
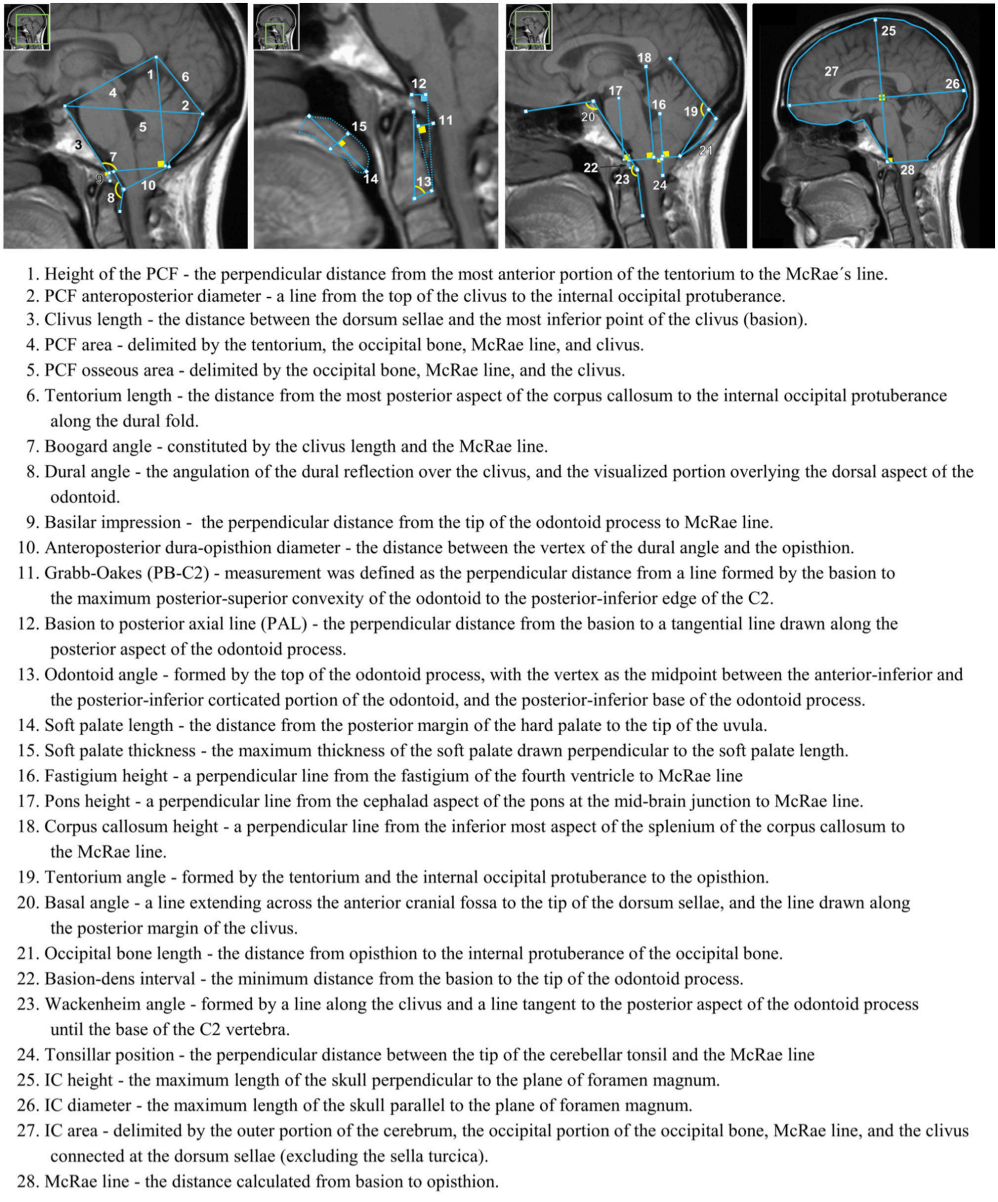
Twenty-eight morphometric measurements taken on a representative midsagittal T1-weighted MRI. Numbers correspond to in-text descriptions.

*MorphPro*, a semi-automated software that was developed in MATLAB (Mathworks, Natick, MA) at The University of Akron, was used to measure 28 soft tissue and bone structures within the PCF compartment, craniocervical junction, oral cavity, and intracranial area using different linear, angular, and area measurements shown in Figure 1. These 28 measurements were selected in a previous study (Houston et al., 2017). Prior to the development of *MorphPro*, morphometric measurements were conducted manually using existing commercial DICOM readers such as OsiriX (Pixmeo SARL, Geneva, Switzerland). *MorphPro* provided the operator with a more efficient method for measuring specific features within a midsagittal image with real-time calculation of lengths, angles, and areas. Additionally, this software provided automatic data recording and storage of all morphometric measurements on the midsagittal image. In a separate study, Houston et al. (2017) demonstrated the reproducibility of results using *MorphPro* through the comparison of identical morphometric parameters measured using OsiriX software.

### Morphometric measurements

Measurement methodology was ratified by a board-certified neuroradiologist (JRB) with experience in neuroimaging and CMI through visual confirmation. Descriptions of all 28 measurements below are previously described in Houston et al. (2017) and are closely related to those described by Urbizu and colleagues (Urbizu et al., 2013, 2014, 2017a). Morphometric measurement number in parenthesis corresponds to number labels in Figure 1.

### Statistical analysis

Morphometric measurement comparisons for 12 PCs were determined using two-tailed independent *t*-tests that compared participants with and without a specific PC. Measurement analysis for each of the 12 PCs was conducted separately (e.g., syringomyelia vs. no syringomyelia) due to the overlap of PC prevalence within the sample. Percentage of multiple conditions within our dataset can be seen in Table 3. To correct for family-wise error, a corrected probability value of 0.002 (0.05/28) was used to determine reliably different measures between the two groups (Tabachnick and Fidell, 2007). All analyses were performed in SPSS 24 (SPSS Inc., Chicago, IL) and Excel (Microsoft, Redmond, WA). For group comparisons where MRI age and/or BMI were found to be significantly different, a one-way analysis of covariance (ANCOVA) was used to control for the effects of age and/or BMI. Controlling for the demographic effects on morphology was motivated by previous findings (see Roller et al., 2015; Houston et al., 2017), which found that differences in morphology were reduced when controlling for demographic variables such as BMI and age. Mann-Whitney *u*-tests were used for group comparisons in which samples did not demonstrate a normal distribution.

**Table 3:** Percentage of CMI sample with more than one condition.

	***N***	**One condition (%)**	**Two conditions (%)**	**Three conditions (%)**	**Four conditions (%)**	**Five conditions (%)**	**Six conditions (%)**	**Seven conditions (%)**
All CMI	236	29.2	28.0	13.6	7.2	1.3	3.4	0.4
Migraine headaches	147	33.3	34.0	15.6	9.5	1.4	5.4	0.7
**Scoliosis**	45	11.1	31.1	37.8	11.1	2.2	4.4	2.2
**Syringomyelia**	38	21.1	28.9	23.7	18.4	2.6	5.3	0.0
Fibromyalgia	33	6.1	27.3	24.2	21.2	6.1	15.2	0.0
Chronic fatigue syndrome	31	3.2	19.4	32.3	25.8	6.5	12.9	0.0
Spinal dysraphism	29	0.0	27.6	20.7	24.1	6.9	17.2	3.4
**Ehlers Danlos syndrome**	21	4.8	23.8	4.8	28.6	0.0	33.3	4.8
Other endocrine diseases	20	0.0	40.0	30.0	15.0	5.0	5.0	5.0
**Pseudotumor cerebri**	20	10.0	15.0	20.0	10.0	10.0	30.0	5.0
Raynaud phenomenon	18	0.0	38.9	22.2	16.7	0.0	22.2	0.0
**Craniocervical instability**	17	5.9	17.6	23.5	29.4	0.0	17.6	5.9
Sleep apnea	16	0.0	50.0	25.0	6.3	12.5	6.3	0.0

*83% of CMI participants have at least one prevalent condition. Conditions in bold are defined as RC*.

## Results

McRae line length was found to be significantly smaller in CMI participants with syringomyelia (Mean_SM_ = 33.8 mm) compared to participants without syringomyelia (Mean_NoSM_ = 35.5 mm), *t*_(234)_ = 3.52, *p* < 0.002, *d* = −0.64. TP was found to be significantly smaller in CMI patients with EDS (Mean_EDS_ = 5.54 mm) compared to those without EDS (Mean_NoEDS_ = 8.37 mm), *t*_(35.4)_ = 4.17, *p* < 0.002, *d* = −0.71. Basion to PAL length was found to be significantly larger in CMI participants with scoliosis (Mean_S_ = 9.11 mm) compared to those without scoliosis (Mean_NoS_ = 7.54 mm), *F*_(1, 201)_ = 10.73, *p* < 0.002, *d* = 0.46. No significant differences were found across pseudotumor cerebri, CCI, CFS, fibromyalgia, Raynaud phenomenon, migraine headaches, spinal dysraphism, endocrine disease, or sleep apnea diagnoses.

**Figure 2:**
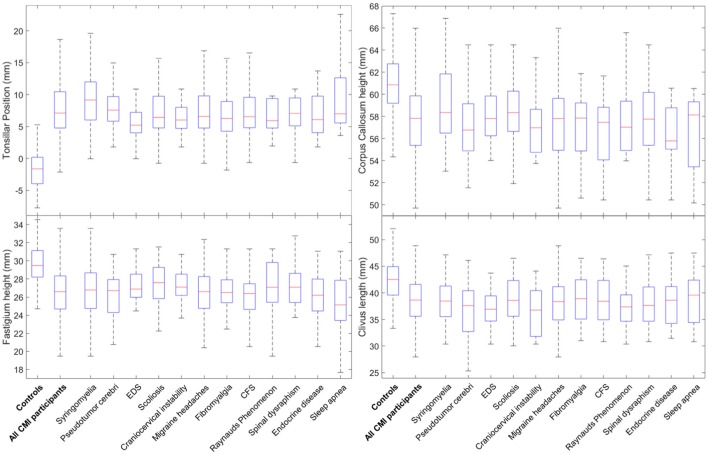
Boxplots for group differences between 12 PCs and controls for tonsillar position, fastigium heights, corpus callosum height, and clivus length. Horizontal lines within boxes represent group median, boxes represent the 25th and 75th percentile rank of group values.

MMs were also compared separately for each related condition against controls. This analysis revealed many differences between CMI participants with specific PCs and controls. For all 12 PCs, group differences between CMI participants and controls were identified for four MMs. TP was significantly larger and fastigium height, corpus callosum height, and clivus length were significantly smaller in CMI participants for all 12 PCs (Figure 2). Pons height was also found to be significantly smaller in eight of the 12 PCs (see Tables 4, 5 for statistical results). Group differences for all five RCs and four of seven APCs showed a smaller PCF osseous area in CMI participants. None of the RCs demonstrated group differences for PCF height or area, or for any of the intracranial measurements when compared to controls. Of the APCs, only migraine headaches showed significant differences in PCF height, PCF area, intracranial height, or intracranial area. Additional morphometric differences between CMI with a specified PC and controls will be discussed below.

**Table 4:** Descriptive statistics for comparison of CMI participants with related conditions and control participants.

**No**.	**Measurements**	**Syringomyelia**		**Pseudotumorcerebi**		**Ehlers-Danlos syndrome**		**Scoliosis**		**Craniocervical instability**	
		**Meandiff**	**Cohen's *d***	**Meandiff**	**Cohen's *d***	**Meandiff**	**Cohen's *d***	**Meandiff**	**Cohen's *d***	**Meandiff**	**Cohen's *d***
28	McRae line length	−0.37	−0.19	1.64	0.42^*^	***2.23***	0.59^**^	1.65	0.59^**^	1.19	0.23
24	Tonsillar position	10.3	2.26^**^	10.1	1.81^**^	7.56	1.61^**^	9.36	2.31^**^	9.30	1.59^**^
16	Fastigium height	−2.5	−0.89^**^	−3.08	−0.84^**^	−2.21	−0.73^**^	−2.42	−0.94^**^	−2.03	−0.59^**^
17	Pons height	−***1.85***	−0.53^**^	−2.38	−0.52^*^	−***1.98***	−0.52*	−1.37	−0.45^*^	−2.91	−0.66^**^
18	Corpus Callosum height	−1.81	−0.58^**^	−3.81	−0.77^**^	−2.13	−0.56^**^	−2.84	−0.82^**^	−2.90	−0.64^**^
3	Clivus length	−3.63	−0.77^**^	−5.16	−0.79^**^	−5.61	−0.96^**^	−3.56	−0.74^**^	−6.08	−0.94^**^
1	PCF height	−0.66	−0.18	−***2.16***	−***0.24***	−0.35	−0.04	−***1.55***	−***0.28***	−1.31	−0.20
4	PCF area (mm^2^)	−***71.5***	−***0.28***	−***162***	−***0.32***	−65.3	−0.15	−***108***	−***0.30***	−***131***	−***0.31***
5	PCF osseous area (mm^2^)	−148	−0.62^**^	−255	−0.67^**^	−198	−0.62^**^	−164	−0.57^**^	−252	−0.71^**^
20	Basal angle (°)	6.52	0.71^**^	7.85	0.59^**^	***8.52***	0.72^**^	***4.46***	0.49*	4.60	0.36^*^
23	Wackenheim angle (°)	−6.37	−0.74^**^	−***4.11***	−0.54*	−***3.69***	−0.54*	−7.55	−0.77^**^	−2.40	−0.33
7	Boogard angle (°)	5.30	0.63^**^	***3.27***	***0.32***	4.80	0.51^*^	3.27	0.42^*^	4.24	0.38^*^
10	AP dura-Op	0.72	0.17	1.22	0.41^*^	1.49	0.46^*^	1.52	0.55^**^	1.22	0.30
12	Basion to PAL	0.64	0.37^*^	2.71	0.78^**^	2.42	0.76^**^	2.955	1.00^**^	***1.45***	0.38*
13	Odontoid angle (°)	−4.13	−0.63^**^	−1.72	−0.23	−3.15	−0.41^*^	−3.57	−0.55^**^	−4.37	−0.53^*^
25	Intracranial height	−1.59	−0.34^*^	−3.01	−0.39^*^	−2.44	−0.37^*^	−2.31	−0.41^*^	−***3.37***	−0.44*
26	Intracranial diameter	−***2.47***	−***0.28***	−***2.98***	−***0.29***	−0.89	−0.08	−1.98	−0.20	−***3.18***	−***0.27***
27	Intracranial area (mm^2^)	−281	−0.26	−***332***	−***0.23***	−168	−0.12	−***309***	−***0.25***	−***446***	−***0.29***

*Measurements in millimeters unless otherwise noted, Meandiff: RC-control. Italicized and bold values show changes in significant and trending results when controlling for age and/or BMI group differences. Measurements highlighted in blue were found to be significantly different in all 12 PCs*.

* *p < 0.05*,

** *p < 0.002*.

**Table 5:** Descriptive statistics for comparison of CMI participants with additional prevalent conditions and control participants.

**No**.	**Measurements**	**Migraine headaches**		**Fibromyalgia**		**Chronic fatigue syndrome**		**Raynaud phenomenon**		**Spinal dysraphism**		**Endocrine disease**		**Sleep apnea**	
		**Meandiff**	**Cohen's *d***	**Meandiff**	**Cohen's *d***	**Meandiff**	**Cohen's *d***	**Meandiff**	**Cohen's *d***	**Meandiff**	**Cohen's *d***	**Meandiff**	**Cohen's *d***	**Meandiff**	**Cohen's *d***
28	McRae line length	1.31	0.52^**^	0.83	0.17	0.80	0.11	0.68	0.1	***0.73***	***0.22***	0.80	0.14	1.92	0.78^*^
24	Tonsillar position	9.64	2.45^**^	9.39	1.96^**^	9.58	1.94^**^	9.51	1.70^**^	8.78	2.05^**^	8.30	1.49^**^	11.25	2.57^**^
16	Fastigium height	−2.96	−1.15^**^	−2.91	−1.02^**^	−2.90	−0.98^**^	−2.65	−0.80^**^	−2.32	−0.86^**^	−3.43	−0.93^**^	−4.35	−1.40^**^
17	Pons height	−2.26	−0.72^**^	−2.55	−0.78^**^	−2.38	−0.70^**^	−2.75	−0.68^**^	−***2.40***	−0.62^**^	−4.35	−0.90^**^	−3.73	−0.97^*^
18	Corpus Callosum height	−3.16	−1.03^**^	−3.34	−0.88^**^	−3.71	−0.95^**^	−3.09	−0.68^**^	−2.86	−0.74^**^	−4.07	−0.83^**^	−4.22	−1.25^**^
3	Clivus length	−3.70	−0.87^**^	−3.24	−0.62^**^	−2.83	−0.54^**^	−4.73	−0.81^**^	−3.97	−0.74^**^	−4.74	−0.70^**^	−3.75	−0.83^**^
1	PCF height	−2.78	−0.64^**^	−2.27	−0.35^*^	−***3.03***	−0.50*	−1.75	−0.23	−0.98	−0.19	−***2.58***	−***0.32***	−2.98	−0.58^*^
4	PCF area (mm^2^)	−144	−0.53^**^	−***132***	−***0.32***	−***193***	−0.48*	−***143***	−***0.33***	−115	−0.33^*^	−***148***	−***0.31***	−230	−0.80^*^
5	PCF osseous area (mm^2^)	−148	−0.60^**^	−***104***	−***0.32***	−124	−0.37^*^	−176	−0.54^*^	−159	−0.55^**^	−248	−0.63^**^	−233	−0.92^**^
20	Basal angle (°)	5.51	0.70^**^	3.35	0.34^*^	3.58	0.36^*^	7.28	0.64^**^	4.21	0.45^*^	4.98	0.32	4.77	0.61^*^
23	Wackenheim angle (°)	−5.92	−0.85^**^	−***4.45***	−0.68^**^	−7.07	−0.86^**^	−8.38	−0.75^**^	−***2.76***	−0.36*	−7.72	−0.71^**^	−1.03	−0.11
7	Boogard angle (°)	4.23	0.62^**^	4.67	0.63^**^	***4.77***	0.61^**^	6.20	0.62^**^	3.47	0.40^*^	8.54	0.73^**^	6.47	0.71^*^
10	AP dura–Op	1.66	0.72^**^	***0.83***	***0.25***	1.59	0.38^*^	0.69	0.17	0.83	0.26	−0.45	−0.02	2.00	0.75^*^
12	Basion to PAL	1.93	0.84^**^	1.81	0.77^**^	1.29	0.51^*^	***2.04***	0.62^**^	1.13	0.39^*^	3.52	0.88^**^	1.13	0.45
13	Odontoid angle (°)	−3.49	−0.58^**^	−1.86	−0.33^*^	−3.94	−0.60^**^	−2.00	−0.32	−2.58	−0.39^*^	−***4.81***	−0.54*	−4.23	−0.96^**^
25	Intracranial height	−2.52	−0.55^**^	−2.3	−0.42^*^	−1.97	−0.35^*^	−2.48	−0.38^*^	−2.99	−0.46^*^	−5.60	−0.66^**^	−2.78	−0.48^*^
26	Intracranial diameter	−***2.67***	−0.36*	−***4.29***	−0.44*	−***5.07***	−0.45*	−1.1	−0.07	−4.01	−0.46^*^	−***3.82***	−***0.32***	−4.74	−0.68^*^
27	Intracranial area (mm^2^)	−387	−0.43^**^	−***474***	−0.38*	−***610***	−0.45*	−232	−0.16	−519	−0.42^*^	−***441***	−***0.27***	−451	−0.48

*Measurements in millimeters unless otherwise noted, Meandiff: APC-control. Italicized and bold values show changes in significant and trending results when controlling for age and/or BMI group differences. Measurements highlighted in blue were found to be significantly different in all 12 PCs*.

* p < 0.05 and

** *p < 0.002. Sleep apnea results could not be controlled for group differences in age or BMI due to small sample sizes*.

### Related conditions

#### Syringomyelia

Within the PCF compartment, CMI participants with syringomyelia demonstrated a significantly reduced pons height. Angular differences demonstrated a wider basal and Boogard angle in addition to a narrower Wackenheim and odontoid angle in CMI participants with syringomyelia compared to controls. McRae line length was not significantly different between CMI syringomyelia participants and controls.

#### Pseudotumor cerebri

Morphometric differences for CMI patients with pseudotumor cerebri compared to controls were identified for basal angle and basion to PAL length, where pseudotumor cerebri participants demonstrated a larger basal angle, and a larger basion to PAL length.

#### EDS

CMI participants with EDS had a significantly larger McRae line length, and basion to PAL length compared to controls. Angular differences showed significantly wider basal angles in EDS participants compared to controls.

#### Scoliosis

CMI participants with scoliosis demonstrated many morphometric differences when compared to controls. McRae line length, anteroposterior dura-opisthion, and basion to PAL, were found to be significantly larger in CMI scoliosis participants. Angular differences for Wackenheim and odontoid angles showed narrower angles in CMI scoliosis participants compared to controls.

#### CCI

In addition to CMI syringomyelia participants, CMI participants with CCI had significantly smaller pons lengths compared to controls, while no significant differences were found for McRae line length.

### Additional prevalent conditions

#### Migraine headaches

CMI participants with migraine headaches showed the largest number of significant morphometric differences when compared to controls. These morphometric differences included a wider McRae line length, AP dura-opisthion, and basion to PAL length in CMI participants with migraine headaches. Additional PCF measurements including pons height, PCF height, PCF area, and PCF osseous area were found to be significantly smaller in CMI participants with migraine headaches. CMI participants with migraine headaches demonstrated angular differences including a wider basal and Boogard angle, and a narrower Wackenheim and odontoid angle. Intracranial measurements reflected a significantly smaller intracranial height and area in CMI participants with migraine headaches compared to controls.

#### Fibromyalgia and CFS

CMI participants with Fibromyalgia and CFS demonstrated similar morphometric differences from controls, including a smaller pons height. A significantly smaller Wackenheim angle and a significantly larger Boogard angle were identified for both fibromyalgia and CFS participants. Morphometric differences identified included a significantly larger basion to PAL length in fibromyalgia participants, while basion to PAL length was found to be trending in CFS participants. Odontoid angle was found to be significantly smaller in CFS participants, with differences trending in fibromyalgia participants.

#### Raynaud phenomenon

Morphometric differences in CMI participants with Raynaud phenomenon were identified for many of the angular measurements including wider basal and Boogard angles, and a narrower Wackenheim angle compared to controls. Basion to PAL length was significantly longer in Raynaud phenomenon participants.

#### Spinal dysraphism

CMI participants with spinal dysraphism showed limited additional group differences including a significantly reduced pons height and PCF osseous area.

#### Endocrine disease

Morphometric differences for CMI participants with endocrine disease were identified as a significantly reduced PCF osseous area compared to controls. Angular differences included a significantly narrower Wackenheim angle and wider Boogard angle in CMI participants with endocrine disease. A larger basion to PAL length, and reduced pons and intracranial height were also found in CMI participants with endocrine disease when compared to controls.

#### Sleep apnea

Morphometric differences for CMI sleep apnea participants and controls included a significantly narrower odontoid angle in CMI participants. Additionally, the PCF osseous area was significantly reduced in sleep apnea CMI participants.

### Etiological factor groups

In addition, we further tested our dataset by combining the many PCs into four groups. Three groups were previously defined by Milhorat et al. (2010) as CMI and cranial settling (CS), CMI and space-occupying intracranial lesions (SIL), and classical CMI. A fourth group was classified as CMI and endocrine diseases (ED) due to the high saturation of participants with ED in our sample. These groups are comprised of the following PCs: CS - EDS, Marfan syndrome, MASS, scoliosis, Klippel Feil syndrome, or CCI; SIL - hydrocephalus or brain tumors; and ED - growth hormone deficiency, abnormal pituitary, or other endocrine diseases. Classical Chiari participants do not have any of the three previously mentioned groupings of PCs.

No significant morphometric differences were observed between CMI participants with and without CS, SIL, or ED. Compared to controls, measurements from all four groups exhibited lower TP, and a smaller fastigium, pons, CC and clivus length. Additionally, a reduced PCF osseous area, a wider basal angle, and a narrower Wackenheim angle were identified compared to controls. MM statistical results of these large groupings can be seen in Table 6.

**Table 6:** Descriptive statistics for comparison of CMI participants when evaluated by Etiological Factor Groups and control participants.

**No**.	**Measurements**	**CMI and cranial settling**		**Space-occupying intracanial lesions**		**Endocrine diseases**		**Classical Chiari**	
		**Meandiff**	**Cohen's *d***	**Meandiff**	**Cohen's *d***	**Meandiff**	**Cohen's *d***	**Meandiff**	**Cohen's *d***
28	McRae line length	1.44	0.55^**^	0.78	0.16	1.01	0.24	1.18	0.44^**^
24	Tonsillar position	9.07	2.37^**^	10.6	1.98^**^	8.72	1.94^**^	10.5	2.46^**^
16	Fastigium height	−2.43	−1.03^**^	−2.43	−0.76^**^	−2.86	−0.94^**^	−3.27	−1.26^**^
17	Pons height	−1.90	−0.65^**^	−2.59	−0.63^**^	−2.96	−0.76^**^	−2.13	−0.68^**^
18	Corpus Callosum height	−2.78	−0.90^**^	−2.61	−0.61^**^	−3.47	−0.88^**^	−2.96	−0.91^**^
3	Clivus length	−4.25	−0.98^**^	−3.60	−0.60^**^	−4.61	−0.84^**^	−3.14	−0.75^**^
1	PCF height	−1.26	−0.27	−1.99	−0.27	−3.57	−0.56^**^	−2.83	−0.62^**^
4	PCF area (mm^2^)	−103	−0.33^*^	−144	−0.32	−190	−0.48^*^	−142	−0.50^**^
5	PCF osseous area (mm^2^)	−187	−0.75^**^	−229	−0.62^**^	−200	−0.64^**^	−121	−0.49^**^
20	Basal angle (°)	5.74	0.67^**^	8.34	0.73^**^	7.15	0.62^**^	4.64	0.62^**^
23	Wackenheim angle (°)	−6.84	−0.74^**^	−7.71	−0.62^**^	−6.20	−0.68^**^	−6.07	−0.86^**^
7	Boogard angle (°)	4.00	0.57^**^	5.49	0.54^*^	4.93	0.52^*^	4.05	0.60^**^
10	AP dura-Op	1.45	0.55^**^	1.90	0.47^*^	0.21	0.12	1.83	0.79^**^
11	Grabb-Oakes	0.47	0.34^*^	−0.35	−0.11	0.76	0.49^*^	−0.14	−0.02
12	Basion to PAL	2.39	0.88^**^	1.71	0.45^*^	2.65	0.85^**^	1.19	0.59^**^
13	Odontoid angle (°)	−3.87	−0.69^**^	−4.78	−0.55^**^	−2.84	−0.42^*^	−3.60	−0.60^**^
25	Intracranial height	−2.34	−0.47^*^	−1.15	−0.22	−3.73	−0.56^**^	−2.05	−0.46^**^
26	Intracranial diameter	−1.59	−0.17	−0.98	−0.07	−3.38	−0.35^*^	−2.55	−0.40^*^
27	Intracranial area (mm^2^)	−277	−0.24	−114	−0.10	−448	−0.33^*^	−326	−0.39^*^

*Measurements in millimeters unless otherwise noted, Meandiff: PC-control. Measurements highlighted in blue were also found to be significantly different in the 12 PCs*.

* *p < 0.05*,

** *p < 0.002*.

## Discussion

This retrospective case-control study of adult female CMI patients (*n* = 236) and age-gender matched controls (*n* = 140) assessed 28 morphometric features in a subject population based on midsagittal MRI scans. Measurements were analyzed with respect to study group and self-reported PCs. Unique morphometric features for each PC were determined by examining PCs which had a high prevalence within our CMI dataset. PCs were evaluated in two ways: (1) MMs were compared between CMI participants with and without a specific PC, and (2) CMI participants were morphometrically compared to controls.

### (1) Comparisons between adult female CMI participants with and without specific prevalent conditions

In our study, few MMs were found to distinguish between CMI participants with and without the examined PCs. McRae line length was the only MM that differed based on syringomyelia status and was smaller in CMI participants with syringomyelia. Additionally, McRae line length was not significantly different in syringomyelia patients than in controls. Previous case-control studies that evaluated both male and female CMI patients showed that McRae line length was larger in CMI patients than in healthy controls (Nishikawa et al., 1997; Milhorat et al., 1999; Karagöz et al., 2002; Aydin et al., 2005; Dagtekin et al., 2011; Houston et al., 2017). These findings suggest that a normal-sized McRae line length is unique to adult women CMI patients with syringomyelia. Additional follow-up studies may be necessary to examine whether CMI adult female patients with a normal McRae line length are at greater risk for syrinx development.

We found TP to be the only MM to distinguish between CMI patients with and without EDS. In contrast to this observation, 71% of the CMI patients diagnosed with HDCT from Milhorat et al. (2007) showed an increased retro-odontoid pannus formation with basilar impression. However, Milhorat et al. (2007) evaluated both male and female CMI patients, while our study only evaluated adult women. Additionally, due to a small occurrence of additional HDCT conditions within our dataset, we could not evaluate the remaining HDCT conditions discussed in Milhorat's et al (2007) study. Discrepancies between results from this previous study and our results may be due to the aforementioned sample differences. A final morphometric distinction was found between CMI patients with and without scoliosis, where basion to PAL length was significantly longer in CMI patients with scoliosis. However, to our knowledge, no prior morphological study has examined similar midsagittal 2D morphometrics in CMI patients with scoliosis. Thus this finding requires further investigation. Aside from syringomyelia, EDS, and scoliosis participants, no other significant morphometric differences were identified for CMI participants with any of the remaining RCs, or APCs in the present study.

When evaluating the etiological factor groups, no significant morphometric differences were found when comparing CMI participants with CS, SIL, or ED to classical CMI participants. Milhorat et al. (2010) compared only groups with CS, SIL and classical CMI participants to healthy controls. However, examination of their results indicated that differences may have been present and thus, motivated this additional test. The cause of these differences across studies is unclear and should be further examined in future studies.

### (2) Comparisons between adult female chiari participants with PC vs. healthy controls

Many MMs within our study were found to distinguish CMI patients with specific PCs from controls including four that were consistently different in all 12 examined PCs. These MM differences included a lower TP (9.5 mm), and shorter clivus, fastigium, and corpus callosum heights that differed by a small margin (2–5 mm). We previously identified similar differences in a subset 302 individuals from the present study (Houston et al., 2017). Eighteen MMs, including the four aforementioned MMs, were significantly different for all CMI participants compared to controls. In the present study, no specific PCs reflected this exact pattern of morphometric differences. This suggests that the four case-control MM differences found across PCs in the present study are the most robust identifiers of CMI. Alternatively, these varying morphometric results could also be attributed to differing demographic characteristics, and smaller samples sizes for the examined PCs.

In addition to these four MMs, CMI participants with EDS had a larger basion to PAL length, and a larger basal angle compared to controls. In a previous analysis of HDCT patients that incorporated EDS, Milhorat et al. (2007) found basion-dens interval and clivus-axial angle (Wackenheim angle) to be significantly different in CMI patients compared to controls. However, our analysis did not show differences in basion-dens interval or Wackenheim angle.

In an analysis of sub-groupings of CMI participants similar to those described by Milhorat et al. (2010), we found that classical CMI participants demonstrated a significantly smaller clivus length compared to controls, a finding that replicates Milhorat et al. (2010). However, no case-control differences were observed for McRae line length. In addition, Milhorat et al. (2010) found no MMs that distinguish between CS or SIL and controls. In contrast, we did find significantly smaller clivus lengths for CS, SIL, and ED participants compared to controls. This discrepancy may be due to the inclusion of male participants, in addition to the MMs being evaluated on CT images in Milhorat et al. (2010). Therefore, by excluding male CMI participants from our MRI analysis, we were able to better identify unique morphology for adult female participants with specific etiological factors.

## Conclusions

From the large number of morphometric measurements evaluated within this study, few were found to differentiate between adult female CMI patients with or without one of the 12 evaluated PCs. MMs which were found to differ within conditions (i.e., between those with a PC and those without the same PC) included McRae line length in syringomyelia, TP in EDS, and basion to PAL length in scoliosis CMI participants. Additionally, while few MMs were found to differentiate between PCs, four morphometric measurements were found to consistently differ from controls regardless of PCs. These MMs included TP, fastigium height, corpus callosum height, and clivus length. This demonstrates inconsistent results from previous case-control studies cannot be accounted for by the saturations of different PCs across study samples. It also suggests that CMI likely manifests similarly in patients whom also contend with various comorbid conditions.

While these results may provide insight into unique morphology related to specific PCs, they are limited because diagnostic information was retrospectively self-reported, and therefore physician diagnostic criteria for the 12 PCs was not guaranteed. Additionally, our sample composition limited our ability to study many characteristics of interest such as: CM patients with single PC, adult men, children, and non-Caucasian patients. Additional approaches, such as three-dimensional structure analysis, brain tissue microstructural analysis, and dynamic measurements of brain motion and/or cerebrospinal fluid dynamics, also remain to be explored. Moreover, 3D morphological analyses may be possible in future investigations using available software for 3D shape analysis such as Deformetrica, Mindboggle, or BrainVISA (Geffroy et al., 2011; Durrleman et al., 2014; Klein et al., 2017).

The results of this morphological analysis may assist future efforts to reveal more complex morphological characteristics of PCs that could improve clinical and pathophysiological understanding of CMI.

## Disclosures

JRB is a recipient of a research grant from the Conquer Chiari. AU was the recipient of a Postdoctoral Fellowship from Fundación Ramón Areces (Spain).

## Ethics statement

This study was carried out in accordance with the recommendations of the review boards of the University of Akron and Akron General Medical Center with written informed consent from all subjects. All subjects gave written informed consent in accordance with the Declaration of Helsinki. The protocol was approved by the review boards of the University of Akron and Akron General Medical Center.

## Author contributions

ME was responsible for software development, data processing, data analysis, and manuscript writing. JH was responsible for data processing, data analysis, and manuscript writing, and project supervision. JB, RL, BM, PA and FL were responsible for project oversight and providing expertise in neuroradiology. DL was responsible for image acquisition, server management, and project oversight. AB and NA were responsible for data processing processing. SH and DB were responsible for software development and data processing. AU was responsible for providing expertise in image analysis. CM was responsible for providing expertise in neuroanatomy.

### Conflict of interest statement

The authors declare that the research was conducted in the absence of any commercial or financial relationships that could be construed as a potential conflict of interest.
